# Supplementary material for: The Significant Role of PA28αβ in CD8+ T Cell-Mediated Graft Rejection Contrasts with Its Negligible Impact on the Generation of MHC-I Ligands
Source: Int J Mol Sci. 2024 May 22;25(11):5649. doi: 10.3390/ijms25115649 (PMC11172216; doi:10.3390/ijms25115649)
Supplement: Supplementary file 1 [file ijms-25-05649-s001.zip › ijms-2982829-supplementary.pdf]

Supplementary Material

The Significant Role of PA28$\alpha\beta$ in CD8$^+$ T Cell-Mediated Graft Rejection Contrasts with its Negligible Impact on the Generation of MHC-I Ligands

Katharina Inholz [1,2], Ulrika Bader [2], Sarah Mundt[3] and Michael Basler [1,2,*]

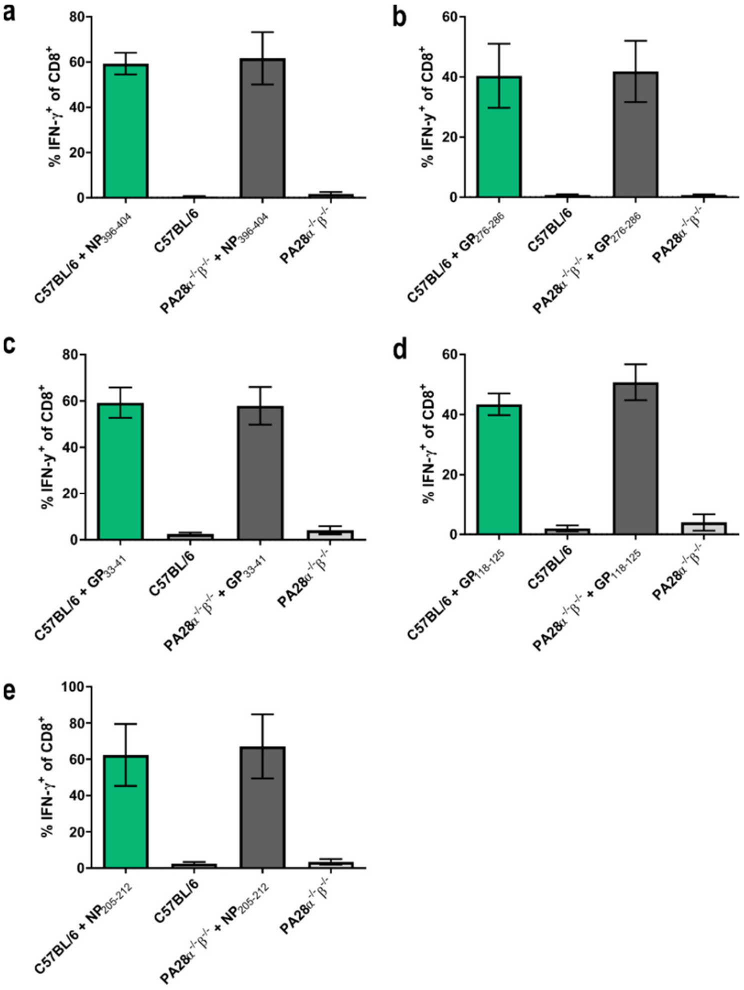

Supplementary Figure S1: (a-e) Uninfected MEFs from either wildtype or PA28$\alpha\beta$ knockout mice were co-cultured with peptide specific CTLs for NP$_{396-404}$ (a), GP$_{276-286}$ (b), GP$_{33-41}$ (c), GP$_{118-125}$ (d), or NP$_{205-212}$ (e) in the presence or absence of the indicated peptides. Activation of CTL-lines was analyzed after five hours of co-culture by staining for CD8 and intracellular IFN-γ. Shown are the percentages of IFN-γ-positive cells of CD8+ cells as determined by flow cytometry. All samples were measured in duplicates. Shown is the mean ± SEM of 3-5 independent experiments with three different preparations of MEFs from wild-type and two independent preparations of MEFs from knockout mice.

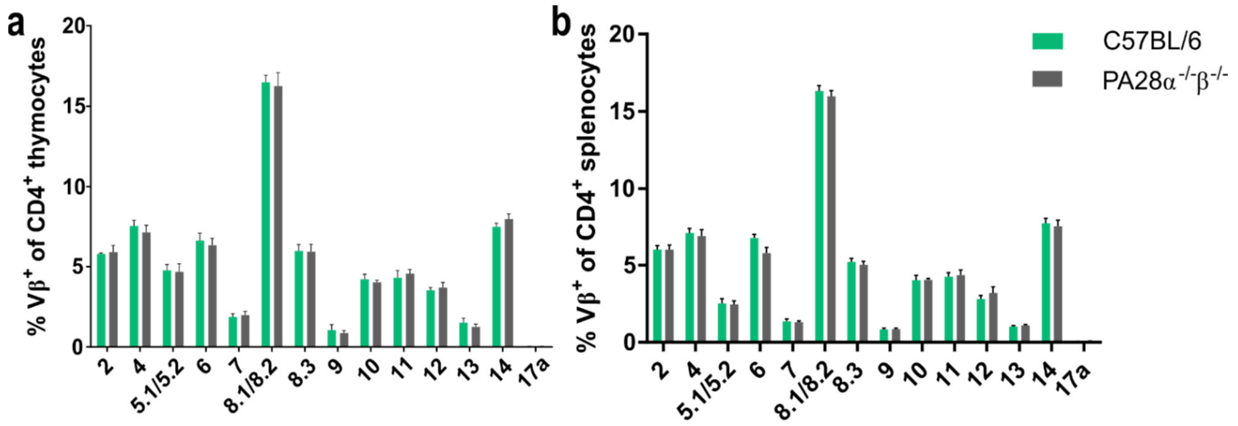

Supplementary Figure S2: Flow cytometric analysis of indicated Vβ variable segments of TCRs from CD4SP thymocytes (a) or splenocytes (b) derived from C57BL/6 mice or PA28αβ-deficient mice. (n = 5 mice per group). V_β17a is not expressed in the C57BL/6 background and was used as negative control. Data are shown as mean ± SEM and were statistically analyzed by Student t test. Unless otherwise specified, the analyses revealed no statistically significant differences.

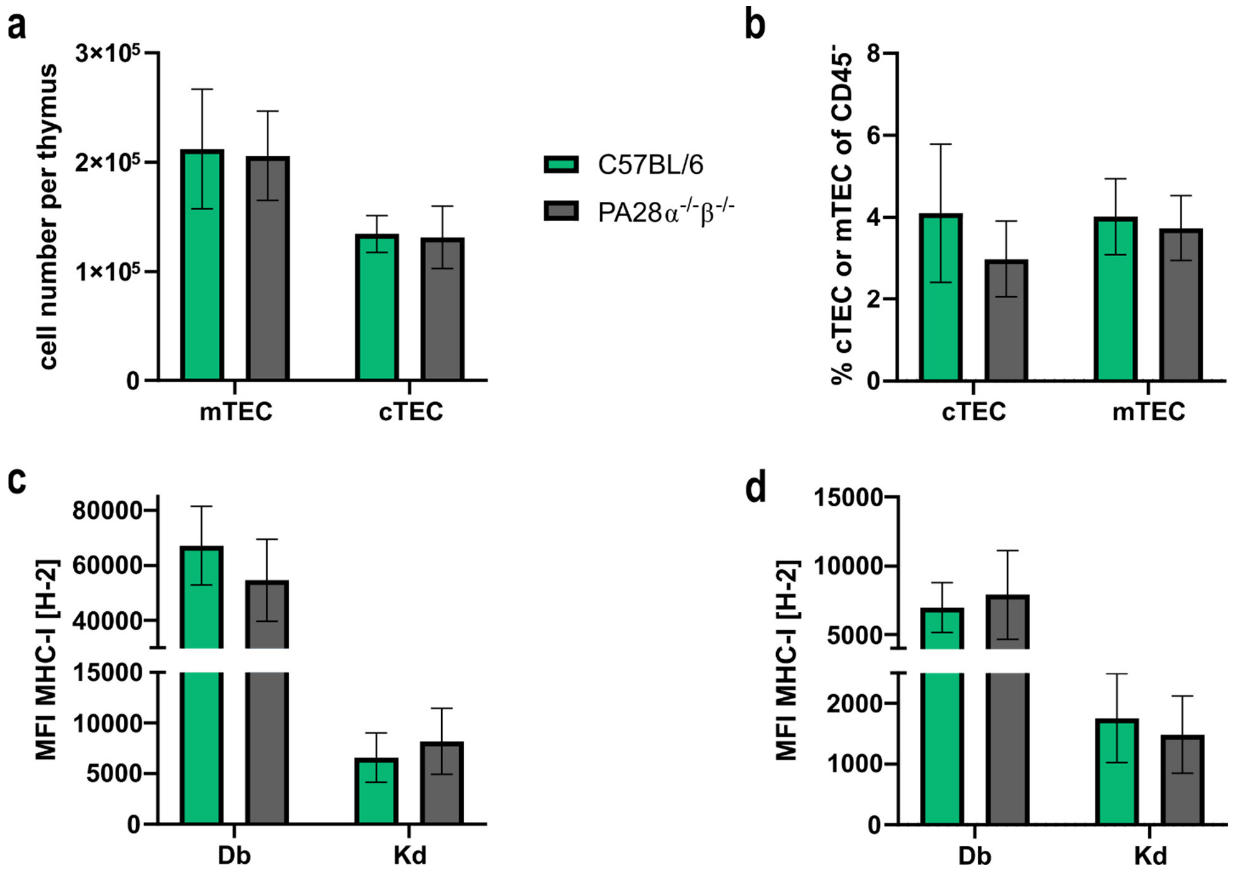

Supplementary Figure S3: Flow cytometry analysis of cTECs (CD45-EpCAM+Ly-51+) and mTECs (CD45-EpCAM+Ly-51-) from wild-type and PA28αβ knockout mice (three to four week-old). **(a)** Total cell number of mTECs and cTECs per thymus. **(b)** Percentage of cTECs and mTECs in total CD45- thymocytes. **(c,d)** Median fluorescence intensity (MFI) of surface MHC class I H-2Kb and H-2Db expression of cTECs **(c)** or mTECs **(d)** from either PA28αβ knockout mice or wild-type mice was measured by flow cytometry. n=5-6 mice per group, measured in duplicates, Data from two independent experiments are shown as mean ± SEM and were statistically analyzed by Student t test. Unless otherwise specified, the analyses revealed no statistically significant differences.

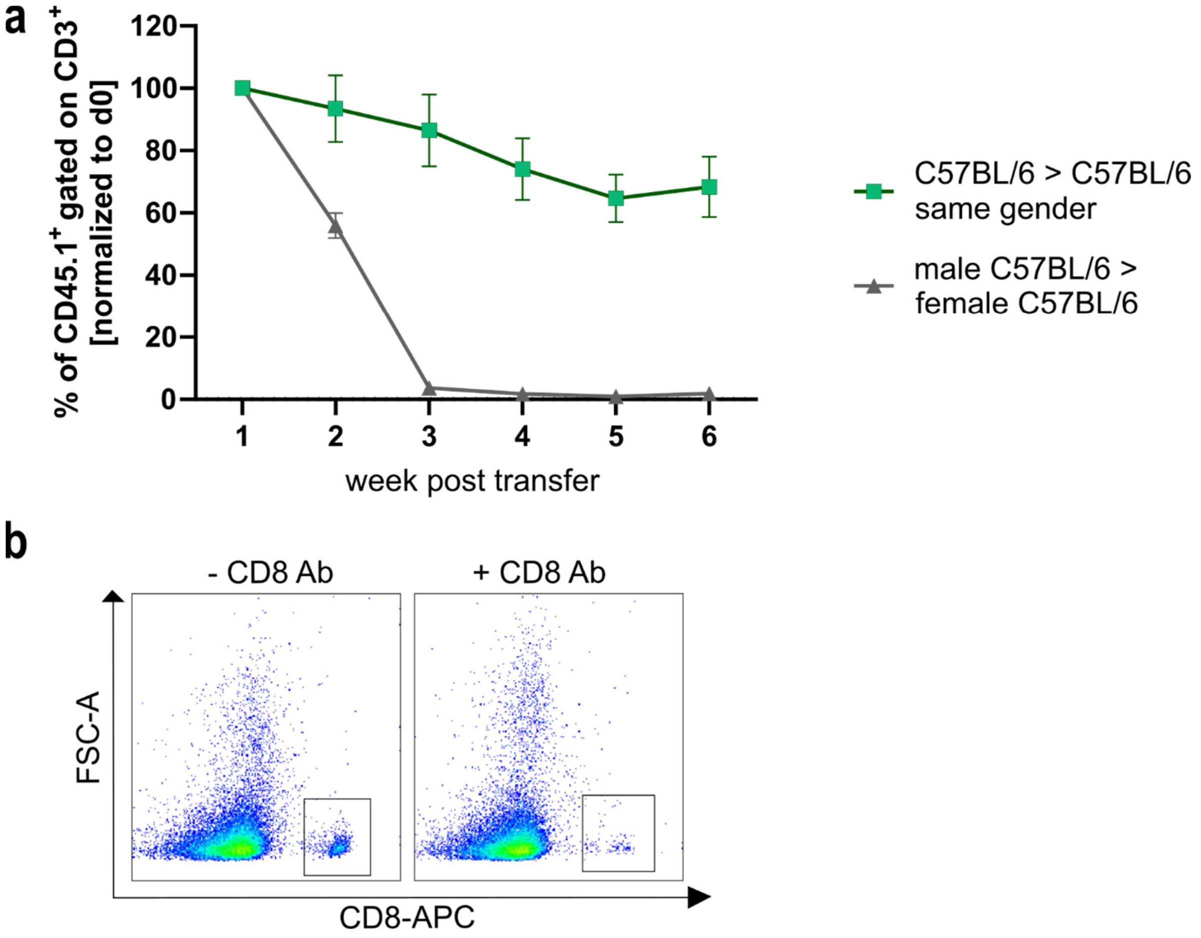

Supplementary Figure S4: (a) Magnetically sorted CD3+ T cells from wildtype (CD45.1+) were transferred into wildtype (CD45.2+) mice of the same gender or magnetically sorted CD3+ T cells from wildtype (CD45.1+) male mice were transferred into wildtype (CD45.2+) female mice. The rejection of the transferred CD3+CD45.1+ cells was monitored weekly in the blood by flow cytometry. **(b)** Magnetically sorted CD19+ B cells were transferred into PA28αβ knockout mice which were either left untreated (left) or treated weekly with an anti CD8+ depleting antibody (right). Depletion of CD8+ T cells was monitored weekly in the blood after staining for CD8 by flow cytometry. The depletion is shown exemplarily.

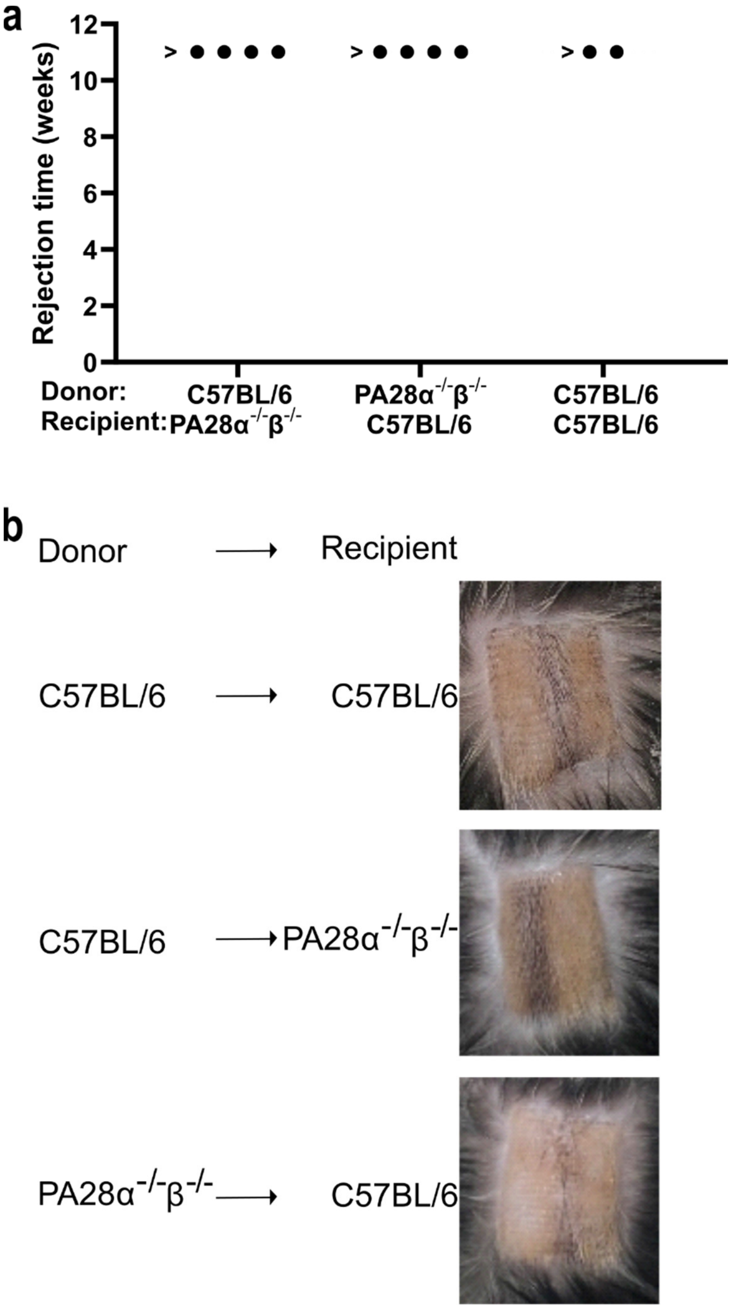

Supplementary Figure S5: Influence of PA28αβ on skin graft rejection. Indicated recipient mice were transplanted with fully-thickness tail skin of C57BL/6 mice or PA28αβ-deficient mice (n=4 or n=2 per group). Graft survival was monitored daily after removal of the bandage. **(a)** Rejection time of skin graft. Experiment was terminated 11 weeks post transplantation. **(b)** representative picture of indicated skin transplants 11 weeks post transplantation.
